# Using a trait‐based approach for assessing the vulnerability and resilience of hillslope seep wetland vegetation cover to disturbances in the Tsitsa River catchment, Eastern Cape, South Africa

**DOI:** 10.1002/ece3.5893

**Published:** 2019-12-11

**Authors:** Notiswa Libala, Carolyn G. Palmer, Oghenekaro Nelson Odume

**Affiliations:** ^1^ Unilever Centre for Environmental Water Quality Institute for Water Research Rhodes University Makhanda (Grahamstown) South Africa

**Keywords:** hill slope seep, livestock grazing, traits, vulnerability or resilience

## Abstract

Hill slope seep wetlands are ecologically and economically important ecosystems as they supply a variety of ecosystem services to society. In South Africa, livestock grazing is recognized as one of the most important disturbance factors changing the structure and function of hill slope seep wetlands. This study sought to investigate the potential effect of livestock grazing on the resilience and vulnerability of hillslope seep wetland vegetation cover using a trait‐based approach (TBA). Changes in vegetation cover were used as a surrogate for indicating grazing intensity. The degree of human disturbances was assessed using the Anthropogenic Activity Index. A TBA was developed using seven plant traits, resolved into 27 trait attributes. Based on the developed approach, plant species were grouped into vulnerable and resilient groups in relation to grazing pressure. It was then predicted that species belonging to the vulnerable group would be less dominant at the highly disturbed sites, as well as in the winter season when grazing pressure is at its peak. The approach developed enabled accurate predictions of the responses of hillslope plant species to grazing pressure seasonally, but spatially, only for the summer season. The predicted responses during the winter season across sites did not match the observed results, which could be attributed to the difficulty in species identification and accurate estimation of vegetation cover during winter. Overall, the approach developed here provides a general framework for applying the TBA and can thus be tested and applied elsewhere.

## Graphical abstract

The trait‐based approach used in this study enables the prediction of the effects of disturbance on vegetation patterns to guide conservation and management efforts. There is an urgent need to develop these approaches as the studied wetlands are disappearing at an increasing rate.

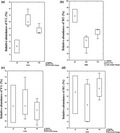

## 1 INTRODUCTION

Hillslope seep wetlands are among the least studied wetlands, yet they are among the most vulnerable wetland systems to disturbances because of their small sizes and steep slope (Ellery et al., 2008). They are typically formed when groundwater flows over an impermeable rock forcing the water to the surface so that on the surface, a hillslope seep is formed (Collins, 2005). Although hillslope seep wetlands are recognized as being important, they are disappearing rapidly because of human disturbances (Roy, Linstrom, & Otto, 2017). In the Tsitsa River catchment in South Africa, hillslope seeps are declining due to disturbances such as poor land‐use management practices and grazing pressure.

Human‐induced disturbances are the most important factors structuring the taxonomic and functional composition of vegetation of wetlands (Bernhardt‐Romermann et al., 2011). In this study, the potential effect of livestock grazing on the resilience and vulnerability of hillslope seep wetland vegetation cover (VC) is investigated. Livestock grazing is known to be a major disturbance altering the condition of wetlands and rangelands, particularly on communal lands (Jones, Fraser, & Curtis, 2010). The impact of livestock grazing on grasslands has been the subject of many studies (Adler, Milchunas, Lauenroth, Sala, & Burke, 2004; Cingolani, Posse, & Collantes, 2005; Dorrough, Ash, & Mcintyre, 2004; Pakeman & Marriott, 2010), but not much attention has been paid to VC resilience and vulnerability in hillslope seep wetland in relation to grazing. Engelhardt and Kadlec (2001) show that plant species losses and changes in diversity can decrease the resilience of ecosystems after a disturbance and can also alter ecosystem functioning.

The biological responses of ecosystems to environmental perturbation depend largely on the individual species traits possessed by the assemblage. Traits play a central role in species–environment relationships because they mediate the responses of species to environmental disturbances (Piliere et al., 2016). Focusing on species traits therefore provides opportunity for understanding the mechanistic relationship between biological responses and the driver of change (McGill, Enquist, Weiher, & Westoby, 2006). The mediation role of traits also implies that they can enable the prediction of the potential responses of biological assemblages to disturbances such as grazing (McGill et al., 2006). Thus, a trait‐based approach (TBA) can help to predict VC responses to human‐induced pressure (Odume, Ntloko, & Akamagwuna, 2018).

Several models on the effect of livestock grazing on plant communities in terms of species traits have been developed to predict plant responses to grazing (Díaz et al., 2007). The generally held hypothesis is that the sensitivity of plant communities to grazing depends on the frequency and strength of plant adaptations to avoid or tolerate herbivory (Díaz, Noy‐meir, & Cabido, 2001; Vesk & Westoby, 2001). This hypothesis predicts that grazing impacts are likely to be minimal in systems where grazing‐resistant traits are well developed and common among plant species, as opposed to systems where such traits are poorly developed or rare (Dubey, Sharma, Raghubanshi, & Singh, 2011). Many of these plant trait models have been developed and applied in terrestrial ecosystems, such as grasslands (de Bello et al., 2010). At the moment, however, very little is known about traits and VC resilience or vulnerability in hillslope seep wetlands. Therefore, the objective of this study is to develop and apply a TBA to assess and predict the potential vulnerability and resilience of hillslope seep plant species and VC to human‐induced disturbances, particularly grazing.

It was hypothesized that species deemed vulnerable to human disturbances based on the developed TBA should be more associated with less disturbed sites compared to sites with more disturbances. It was further hypothesized that grazing pressure on hillslope seeps is higher during the dry season compared with the wet season, and thus VC was expected to decline in the dry season in relation to the wet season.

## 2 STUDY AREA DESCRIPTION

**Figure 1:**
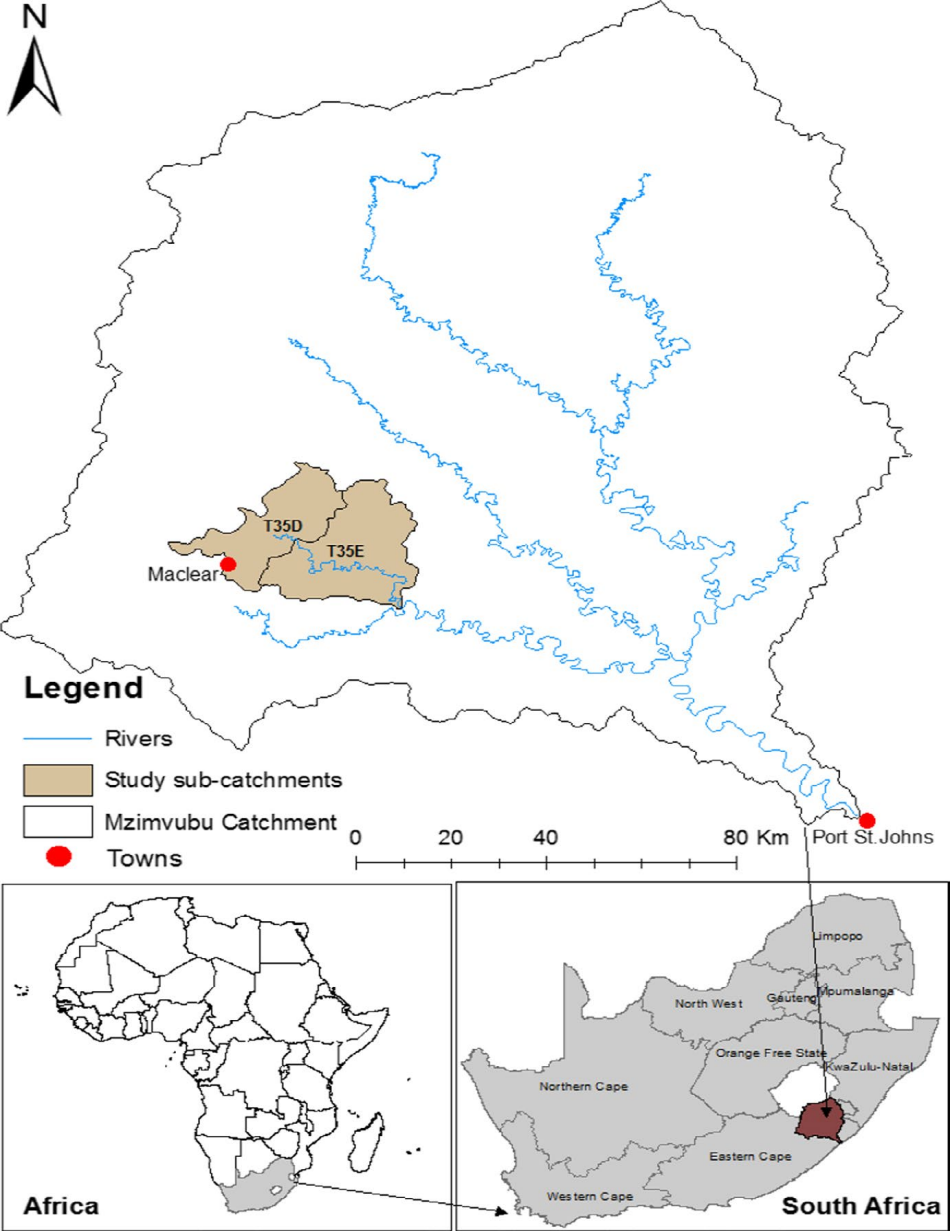
Locality map of quaternary catchments T35D and T35E in the Tsitsa River catchment within the Mzimvubu catchment, Eastern Cape, South Africa

**Figure 2:**
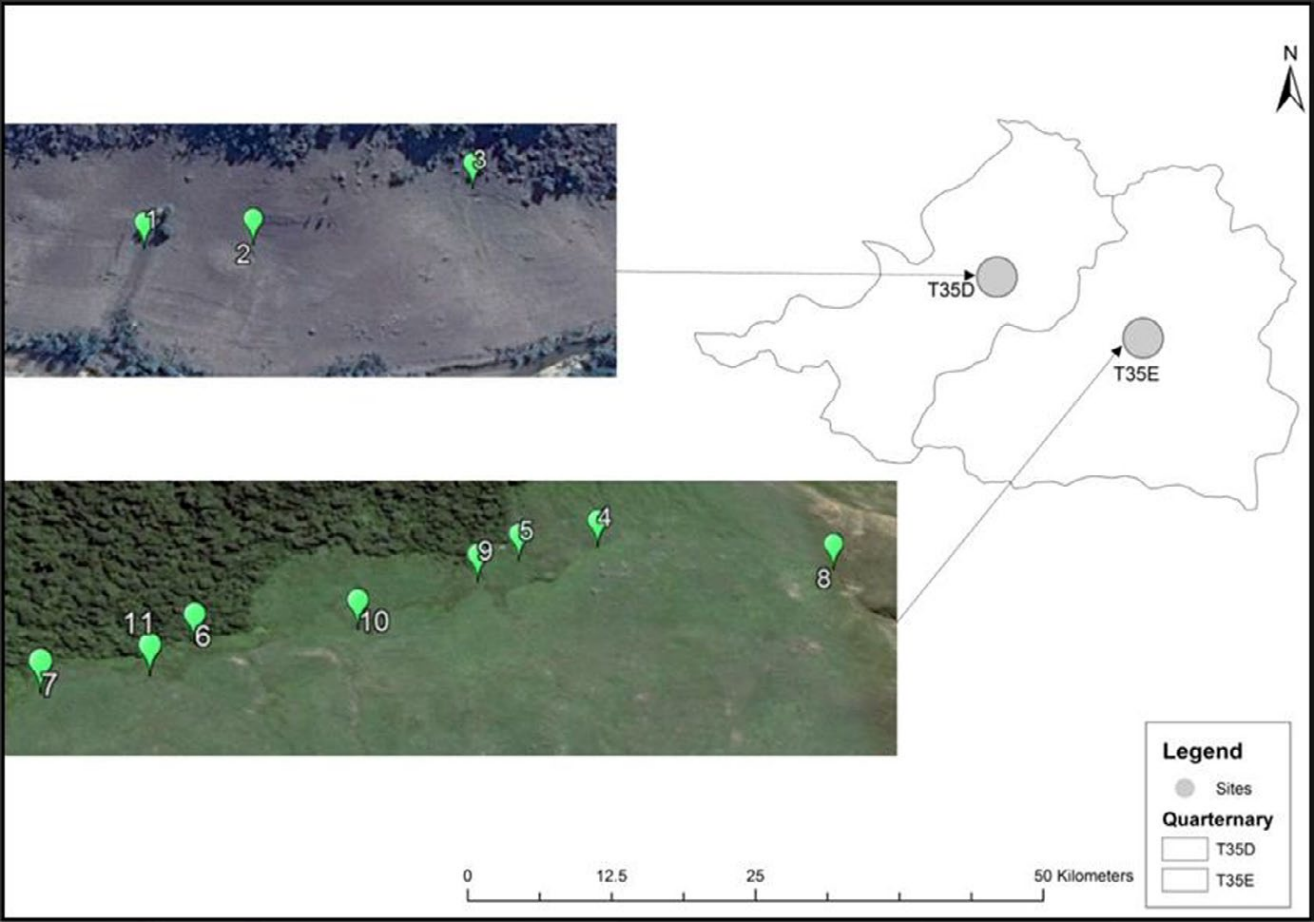
Locality map of selected hillslope seep sites in quaternary catchment T35D and T35E in the Tsitsa River catchment

The study was conducted in the Tsitsa River catchment situated within the upper Mzimvubu catchment in the Eastern Cape Province of South Africa (Figure 1). A total of 11 hillslope seep wetlands were selected for the study. The wetlands were selected subjectively taking into account biophysical factors such as slope aspect, soils and geological characteristics and the degree of erosion, which was visually assessed (Table 1). Three less eroded (LE1, LE2, LE3) hillslope seep wetlands were selected in the T35D quaternary catchment on privately owned land (Figure 2). Eight hillslope seep wetlands situated within a communal grazing area were selected in T35E. Four of the hillslope seeps were moderately eroded (ME1, ME2, ME3, ME4) and four highly eroded (HE1, HE2, HE3, HE4) (Figure 2). Hillslope seep wetland size ranges between 0.05 and 1.2 ha, and their mean elevation ranges from 1,138 to 1,243 m. The catchment is broadly divided into two distinct socio‐cultural domains: the western areas were dominated by freehold title tenure; and the eastern sections, communal areas (Sigwela, Elbakidze, Powell, & Angelstam, 2017). The freehold title areas were characterized by the combined land uses of commercial agriculture and plantation forestry, while the communal areas were characterized by subsistence farming, which includes both livestock and crop production (Van Tol, Akpan, Kanuka, Ngesi, & Lange, 2014). The average rainfall varies from 625 to 1,415 mm per annum (Le Roux, Barker, Weepener, & Berg, 2015), with the maximum rainfall occurring in summer and minimum rainfall in winter. Temperatures range from an average of 14°C in winter to an average of 25°C in summer (Pretorius, 2016). In the winter season, snow is common at the higher altitudes (Sigwela et al., 2017). The geology within the catchment consists of sedimentary shales, mudstones, and sandstones of the Tarkastad subgroup and Beaufort Karoo super group, with the presence of some dolerite intrusions (Blackhurst, Spinks, & Rossouw, 2002). Vegetation in the catchment is classified as subescarpment grassland and subescarpment savanna bioregions dominated by moist grasslands and Acacia spp (Mucina, Rutherford, Phillips, & Rutherford, 2006).

**Table 1:** Visual method used for estimating the degree of erosion of the studied hillslope seep wetland in the current study (adapted from Bunning, McDonagh, & Rioux, 2011)

Erosional category	Description
Low	A few shallow (<0.5 m depth) gullies affecting no more than 5% of the surface and the vegetation cover are good with little soil exposure.
Moderate	Presence of shallow to moderately deep gullies (0.5–1.0 m depth) and/or gullies affecting 5%–25% of the surface area and plant cover is moderate with small bare patches.
High	Presence of deep gullies (>1 m depth) and/or affecting > 25% of the surface and plant cover is very sparse with large bare areas.

## 3 METHODS

### 3.1 Developing a TBA for assessing the vulnerability VC to livestock grazing in hillslope seep wetlands

The capacity to predict vegetation responses to disturbances, such as grazing, requires an understanding of the mechanistic relationship between plant–environmental disturbances, mediated by traits (Lavorel & Garnier, 2002). The approach followed in this study was largely adapted from Odume et al. (2018) and follows five steps in classifying plant species into vulnerability groups:

1. Reviewing the literature for reported grazing modes of stress on plant species;
2. On the basis of the reported grazing modes of stress, select, and measure traits that are mechanistically linked to the modes of stress;
3. Identifying plant traits deemed mechanistically linked to disturbance factors, such as livestock grazing;
4. Identifying vulnerable trait attributes from the selected trait categories;
5. Grouping plant species into three vulnerability groups, based on the combination of plant traits.

#### 3.1.1 Reviewing the literature for grazing modes of stress on VC

Grazing is a complex process that has several effects on plants (Xu et al., 2013). The impact of livestock grazing was reviewed in this study, indicating that the impact on vegetation follows direct and indirect pathways. As reflected in Table 2, direct impacts are related to trampling, plant biomass removal and soil compaction, while indirect impacts are related to shifts in species composition.

**Table 2:** A summary of modes by which grazing impact on plant communities

Grazing mode of stress	Impacts on vegetation
Trampling	Trampling of plant by livestock is common in wetland ecosystems and it negatively impacts on ecosystem functioning, leading to a reduction in vegetation cover and degradation of plant communities (Pescott & Stewart, 2014). Trampling has a direct mechanical effect on plants by causing physical damage through either excess flexural loading or by crushing plant organs (Sun & Liddle, 1993). Species have differential responses to trampling and the impact of trampling differs among individual plant traits. Smaller leave sizes and shorter plants are likely to be more resilient to trampling compared with broad leaves and taller plant species (Sun & Liddle, 1993). Trampling may also compact the soil surface, rendering it more susceptible to runoff and erosion (Dunne, Western, & Dietrich, 2011).
Removal of plant biomass	Grazing may reduce plant biomass and leads to bare patches. Bare ground in wetlands increases soil erosion and accelerates water runoff which increases the amount of soil particles entering the water column (Morris & Reich, 2013). The reduction in plant biomass may provide opportunities for unpalatable species to replace palatable ones (Collins, 2005). The removal of biomass can reduce the amount of water infiltrating the soil which can lead to reduced plant growth. All these factors may in turn have negative impact on wetland condition.
Shift in vegetation communities	The shift in vegetation communities is an indirect effect of grazing. This may occur due to overgrazing or trampling that may allow changes from the dominance of palatable grasses and forbs toward dominance by unpalatable forbs and weedy annuals (McIntyre & Lavorel, 2001).

#### 3.1.2 Trait selection and measurement

The selected plant traits were those that were deemed mechanistically linked to livestock grazing disturbances. Seven plant traits resolved into 27 trait attributes were selected. The seven plant traits were measured according to the standardized world‐wide protocol described by Cornelissen et al. (2003) and included plant height, specific leaf area, palatability, leaf size, leaf dry‐matter content (LDMC), longevity (years), and resprouting potential (Table 3).

**Table 3:** Plant traits, trait attributes, vulnerable trait attributes, and rationalized relationship of the vulnerable trait attributes to grazing stress

Trait category	Trait attribute	Vulnerable trait attribute(s)	Rationale
Plant height (cm) (Díaz et al., 2001)	Short (<40) Medium (40–80) Tall (>80)	Medium–tall (≥40)	Plant height is hypothesized to decrease under increased grazing pressure and taller species, which are within the active grazing height zone of livestock tend to be grazed more than shorter species out of the active grazing zone of livestock, particularly cattle. Shorter plant species therefore tend to dominate in response to grazing (e.g., Díaz et al., 2007; Díaz et al., 2001; Dubey et al., 2011). Cingolani et al. (2005) suggest that shorter plants have a higher growth rate, growing quickly after disturbances, and are more tolerant of herbivory than taller plants. Therefore, taller plant species were deemed more vulnerable to grazing than shorter plant species (Maire, Gross, Pontes, & da, S., Picon‐Cochard, C., & Soussana, J.F., 2009).
Life cycle (years) (Dubey et al., 2011)	Annual Perennial	Perennial	Perennial plants develop more extensive root systems to support their longer lives. However, when grazed heavily, they usually take longer to re‐establish than annual and biannual species (Díaz et al., 2007). On the other hand, annual plant species, being short‐lived and mostly opportunistic with a high relative growth rate, are more resilient to herbivory (Vesk et al., 2004). Grazing thus promotes annual over perennial species (Dubey et al., 2011).
Palatability (Dubey et al., 2011)	Highly palatable Moderately palatable Unpalatable	Highly palatable	Generally, livestock such as cattle and sheep selects the most palatable plant species and avoids species that are difficult to digest (unpalatable) (Grime et al., 1996). Therefore, the impact of grazing on highly palatable species is expected to be higher than that on moderately palatable and unpalatable plant species (Dubey et al., 2011; Vesk & Westoby, 2001).
Specific leaf area (mm^2^/mg) (Walker, Kinzig, & Langridge, 1999)	SLA 1 (<4) SLA 2 (4–8) SLA 3 (8–12) SLA 4 (12–16) SLA 5 (>16)	SLA 1 (< 4)	Specific leaf area (SLA) is an important leaf trait that integrates plant investment into growth vs. defense (Hodgson, Wilson, Hunt, Grime, & Thompson, 1999). Grazing favors species with high SLA and low leaf toughness (Cingolani et al., 2005; Díaz et al., 2001). A high SLA may provide an advantage under heavy grazing because plant species with a high SLA trait turn over leaves rapidly and regrow quickly after grazing (Westoby, 1999). Therefore, species with low SLA tend to be more vulnerable to grazing than those with a high SLA.
Leaf size (mm^2^) (Willby, Abernrthy, & Demars, 2000)	LS1 (<10) LS2 (10–200) LS3 (200–1,000) LS 4 > 1,000	LS 4 > 1,000	Leaf size is a one‐sided, projected surface area of a single or an average leaf expressed in mm^2^. Larger leaves provide better bites for grazers, whereas smaller leaves require more bites in a given leaf (and mass) (Vesk et al., 2004). Plant species with larger leaves are therefore likely to be more attractive to livestock than species with smaller leaves and are therefore more likely to be vulnerable.
Resprouting potential (Cornelissen et al., 2003)	0—never resprouting 20—very poor resprouting 40—moderate resprouting 60—substantial resprouting 80—abundant resprouting 100—very abundant resprouting (these are subjective numbers assigned to species for resprouting capacity after disturbance).	0—never resprouting 20—very poor resprouting	Resprouting refers to the capacity of plants to regenerate from disturbances after damage to the living tissues (Pausas et al., 2016). Resprouting is an important trait for species persistence in an ecosystem with an episodic disturbance regime (Cornelissen et al., 2003). Resprouters survive and accumulate additional belowground biomass through multiple disturbances, and thus their roots are frequently older and larger than those of nonresprouters (Pausas et al., 2016).
Leaf dry‐matter content (mg/g) (Pérez‐Harguindeguy et al., 2013)	LDMC 1 (<150) LDMC 2 (150–300) LDMC 3 (300–500) LDMC 4 > 500	LDMC 1 (<150)	Leaves with high LDMC tend to be tough and thus assumed to be more resistant to livestock grazing than leaves with low LDMC. Species with low LDMC tend to be associated with productive, often highly disturbed environments (Pérez‐Harguindeguy et al., 2013).

Specific leaf area (SLA) was calculated by measuring an area of a fresh leaf divided by its oven dry mass, mm^2^ mg^–1^. At least two fully expanded, hardened, healthy, and light‐exposed leaves per individual plant species were intentionally collected. The purpose was to sample leaves with minimal symptoms from pathogens so that they will give a clear picture when scanned through the leaf area meter. Samples were wrapped in a moist paper and then placed in sealed plastic bags. The collected samples were stored in a cooler box in the field and measured in the laboratory within 24 hr of collection. The collected samples were scanned, and their leaf areas calculated using a leaf area meter. After measuring the fresh area, each leaf sample was placed in an oven at 60°C and left for at least 72 hr to measure the dry weight. Leaf dry‐matter content is an oven dry mass (mg) of a leaf divided by its water‐saturated fresh mass (g) expressed in mg/g. Values of LDMC were calculated as the ratio of the leaf dry mass to the saturated fresh mass (mg/g). Plant height was measured from the base of the stem to the tip of the highest leaf using a tape measure or a meter rule. Palatability and longevity were literature derived (Cornelissen et al., 2003; Dubey et al., 2011; Pausas et al., 2016; Vesk & Westoby, 2001). Resprouting potential was derived from the literature (Cornelissen et al., 2003). Resprouting potential numbers, ranging from 0 to 100, were assigned to species according to the literature. These are arbitrary numbers, where 0 means never resprouting and 100 highly resprouting.

#### 3.1.3 Identifying vulnerable trait attributes from the selected trait categories

Trait attributes likely to confer vulnerability on the species in the context of grazing were identified and termed “vulnerable trait attributes” following the approach developed by Odume et al. (2018). Vulnerable trait attributes were described as trait features possessed by a plant species that increase the plant's likelihood of being vulnerable to a particular environmental stressor (Odume et al., 2018). The identification of vulnerable trait attributes was largely based on the predicted responses of specific trait attributes to grazing in the literature (e.g., Díaz et al., 2007; Díaz et al., 2001; Dubey et al., 2011; Vesk, Leishman, & Westoby, 2004).

#### 3.1.4 Classifying species into vulnerable and resilient groups

Species were classified into two groups (vulnerable and resilient) using percentile distributions of the number of vulnerable trait attributes possessed, the rationale being that species possessed a higher number of vulnerable trait attributes are likely to be more vulnerable than species with fewer vulnerable traits attributes. Species falling ≥60th percentile mark of the number of vulnerable trait attributes were categorized as vulnerable, while those falling <the 60th mark of the number of vulnerable trait attributes were deemed resilient.

#### 3.1.5 Predictions using the percent relative abundance of the vulnerability groups and VC

Using percent relative abundance data for the two groups, it was predicted that species designated as vulnerable would be less frequently associated with the highly disturbed sites (e.g., HE 1, HE 2, HE 3, and HE 4) and the season of intense grazing (winter) than the resilient group. It was also predicted that during period of intensive grazing (dry season), the grazing impact would be higher on seep wetlands and thus the VC was expected to decline compared with wet season.

### 3.2 Disturbance gradient

Vegetation cover was used as a surrogate measure for grazing intensity within each hillslope seep wetland. Direct grazing measurement could not be taken but, given that the hillslope seeps were situated in a rural catchment where no other major activity impact VC apart from grazing, this measure was deemed appropriate. A 100 m transect was established at the center of each seep wetland in order to avoid the possibility of sampling terrestrial plant species. This is particular important because of the very small sizes of hillslope seep wetlands, and thus care was taken not sample terrestrial plant species. However, while care was taken to avoid sampling terrestrial plant species, the sampling strategy deployed may also have led to under sampling and estimation of edged species. Nevertheless, similar sampling strategies have been deployed by (Wardrop, Brooks, Bishel‐Machung, Cole, & Rubbo, 2004). Each transect was marked with small steel pegs so that they could be accurately located in the next sampling season. The vegetation in each site was sampled in two ways. First, the cover was determined following a nondestructive method of Flombaum and Sala (2007). Five (0.2 × 1 m) quadrats were placed along each transect at intervals of 20, 40, 60, 80, and 100 m. In each quadrat, species relative cover and total percentage VC were visually estimated based on the quadrat area covered by grass using categories shown in Table 4. Second, all the vascular species were identified and recorded along the transect to determine species composition using the step‐point method (Evans & Love, 1957). To reduce potential bias from using cover classes, mid‐point of each class was used to estimate VC. The mid‐point values are provided in Table 4.

**Table 4:** Scale used to determine the vegetation cover during the present study (DAFF, 2014)

Categories	% Cover	Mid‐point	Explanation
1	1–10	5.5	Plant cover is very sparse with large bare areas
2	10–25	18	Cover is sparse with some bare areas
3	25–50	38	Cover is moderate with small bare patches
4	50–75	63	Cover is good with only a little soil exposure
5	75–100	88	Cover is dense with no soil visible

#### 3.2.1 Measure of general disturbance gradient

The Anthropogenic Activity Index (AAI) was used to quantify general anthropogenic disturbance in the studied hillslope seep wetlands. The index consists of five metrics, (a) surrounding land‐use intensity, (b) soil disturbance, (c) hydrology and habitat alteration, (d) vegetation community, and (e) habitat alteration within the wetland. The five metrics were assessed at each wetland, scored and then aggregated to provide an AAI value per site. AAI value ranges between 1 and 15, where scores 1–5 indicate least disturbance, 6–10 moderate disturbance, and >10 high disturbance (Ervin, Herman, Bried, & Holly, 2006).

## 4 STATISTICAL AND DATA ANALYSIS

### 4.1 Assessing the predicted responses of plant species to disturbances

To assess the predicted responses of plant species to disturbances, the relative abundance of plant species belonging to groups vulnerable and resilient was regressed against the AAI and VC using a linear regression analysis. AAI and VC were used as explanatory variables, whereas the VG and RG as response variables. The AAI was used as explanatory variable to vulnerability because it integrates physical drivers of biological change, while VC was only used for grazing intensity. The assumptions of normality and homogeneity of variance were investigated using the Shapiro–Wilk test and the Levene's test, respectively. When it appeared that assumptions were violated, data were transformed logarithmically or normalized if assumptions were still not met. Linear regression analyses were undertaken using STATISTICA software package version 13.3.

Boxplots were also used to visualize the distribution of the relative abundances of the VG and RG across the three site groups (i.e., the less eroded, moderately eroded, and highly eroded site groups). Box plots enable visualization of summary statistics such as median, interquartile ranges, and outliers. The Kruskal–Wallis multiple comparison tests were used to test for significant differences (*p* ≤ .05) between the site groups in terms of the relative abundances of the VG and RG.

### 4.2 Assessing differences between the sites and seasons in terms of AAI and VC

A two‐way ANOVA was used to test for differences (*p* < .05) in the means of the AAI and VC between the sites and seasons. When ANOVA indicated significant differences, a post hoc test was used to indicate the sites and season that differed. Prior to using ANOVA, the basic assumptions of normality and homogeneity of variance were investigated using the Shapiro–Wilk test and the Levene's test, respectively. When it appeared that assumptions were violated, data were transformed logarithmically or normalized if assumptions were still not met. ANOVA and Turkey's honestly significant difference (HSD) test were undertaken using the STATISTICA software package version 13.

### 4.3 Association between species and the site groups

Pearson's point‐biserial multivariate correlation coefficient was run to determine the strength and statistical significance of the relationship between species and the site groups (De Caceres, Legendre, Wiser, & Brotons, 2012). Pearson's point‐biserial correlation is a multivariate analysis that concurrently correlates multiple species in relation to the site groups. The purpose of this analysis was to check whether species designated as vulnerable were less associated with the highly disturbed sites than sites with fewer disturbances. For this analysis, the significance of associations was tested using 999 random permutations (*p* < .05). The Pearson point‐biserial analysis was carried out using the “indicspecies” package for R version 3.5.1 (De Caceres et al., 2012; R Development Core Team, 2014).

## 5 RESULTS

### 5.1 Seep wetland disturbances gradient across sites and seasons

**Figure 3:**
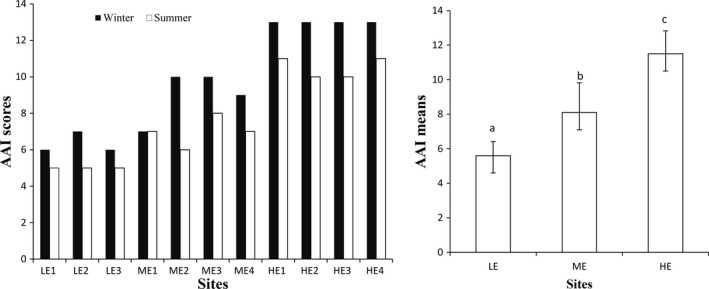
Anthropogenic Activity Index (AAI) showing disturbances gradient across the sites per season (left) (LE = less eroded site, ME = moderately eroded sites, HE = highly eroded sites) and the means ± standard errors of AAI (right). Different small alphabet letters on the bars across the site groups indicate significant differences (*p* < .05), whereas the same letters across sites group indicate no significant differences by ANOVA and Tukey's honestly significant difference test (*p* > .05)

Anthropogenic Activity Index was consistently higher in the highly eroded sites compared with less eroded sites. With regard to season, AAI was higher in the winter season compared with summer (Figure 3). Two‐way ANOVA, followed by the Tukey's HSD post hoc test showed that the mean AAI differed significantly between all the three site groups and between the two seasons (*p* < .05). AAI was significantly higher in the highly eroded sites and in winter season indicating that these sites and season presented more disturbances to hillslope seep wetland (Figure 3).

**Figure 4:**
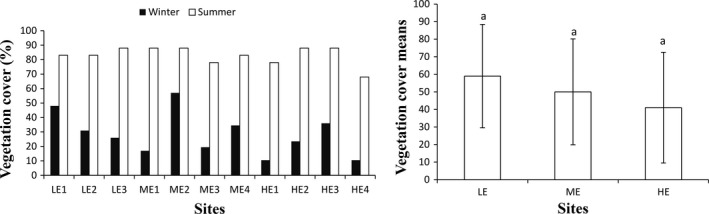
Vegetation cover (VC) across the sites per season (LE = less eroded site, ME = moderately eroded sites, HE = highly eroded sites) on the left and the means ± standard errors of VC on the right.). Different small alphabet letters on the bars across the site groups indicate significant differences (*p* < .05), whereas the same letters across sites group indicate no significant differences by ANOVA and Tukey's honestly significant difference test (*p* > .05)

Vegetation cover was slightly higher in the less eroded sites compared with highly eroded sites. With regard to season, VC was low in the winter season compared with the summer season. Two‐way ANOVA, followed by Tukey's HSD post hoc test, showed that the mean VC did not differ significantly (*p* > .05) between all the three site groups although there was significant differences between the two seasons (*p* < .05) (Figure 4).

Comparing grazing level using VC in the privately owned subcatchment, the results showed that there was no observed trend across sites (LE 1, LE 2, and LE 3) in summer, while in winter, LE 1 site had the highest VC. In the communal subcatchment, the results also showed no differences across the eight sites, while in winter, two of the highly eroded sites had the low VC compared with other sites.

### 5.2 Grouping of plant species according to their potential vulnerability to disturbances

A total of 32 plant species were collected, identified, and classified according to their potential vulnerability to grazing. Of the 32 species recorded, 14 were classified as vulnerable, while 19 were resilient (Table 5). The vulnerable species include *Themeda triandra*, *Hemarthria altissima*, and *Digitaria erientha*. *Richardia brasiliensis*, *Centella asiatica*, and *Eragrostis plana* etc were classified as resilient species (Table 5).

**Table 5:** Plant species grouped according to their potential vulnerability to grazing using the trait‐based approach developed

Resilient	Vulnerability
*Alepidea amatymbica*	*Conyza scabrida*
*Alloteropsis semialata*	*Digitaria erientha*
*Berkheya* sp.	*Hemarthria altissima*
*Centella asiatica*	*Hypoxis acuminata*
*Commelina africana*	*Knowltonia bracteata*
*Cymbopogon validus*	*Mentha aquatica*
*Eragrostis aspera*	*Miscanthus capensis*
*Eragrostis curvula*	*Panicum maximum*
*Eragrostis plana*	*Paspalum dilatatum*
*Gerbera viridifolia*	*Paspalum distichum*
*Haplocarpha lyrata*	*Senecio coronatus*
*Helichrysum aureonitens*	*Senecio speciosus*
*Helichrysum nudifolium*	*Taraxicum officinale*
*Ornithogalum* sp.	*Themeda triandra*
*Polygonum* sp.	
*Richardia brasiliensis*	
*Tristachya hispida*	
*Wahlenbergia* sp.	

### 5.3 Predicted response of vulnerable groups to disturbances

**Figure 5:**
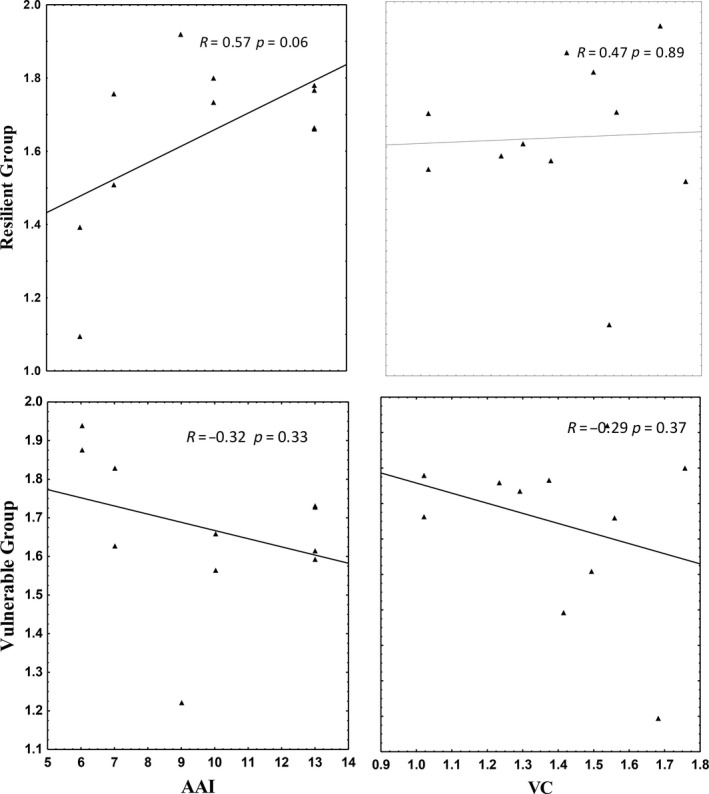
Linear regression between the relative abundance of the vulnerable group species, resilient group species and Anthropogenic Activity Index (AAI) and vegetation cover for the 11 surveyed hillslope seep wetland during winter

The results indicated that during winter, the relative abundance of species designated as vulnerable decreased with increasing AAI, and VC, but the relationship was not statistically significant for either AAI or VC (Figure 5).

**Figure 6:**
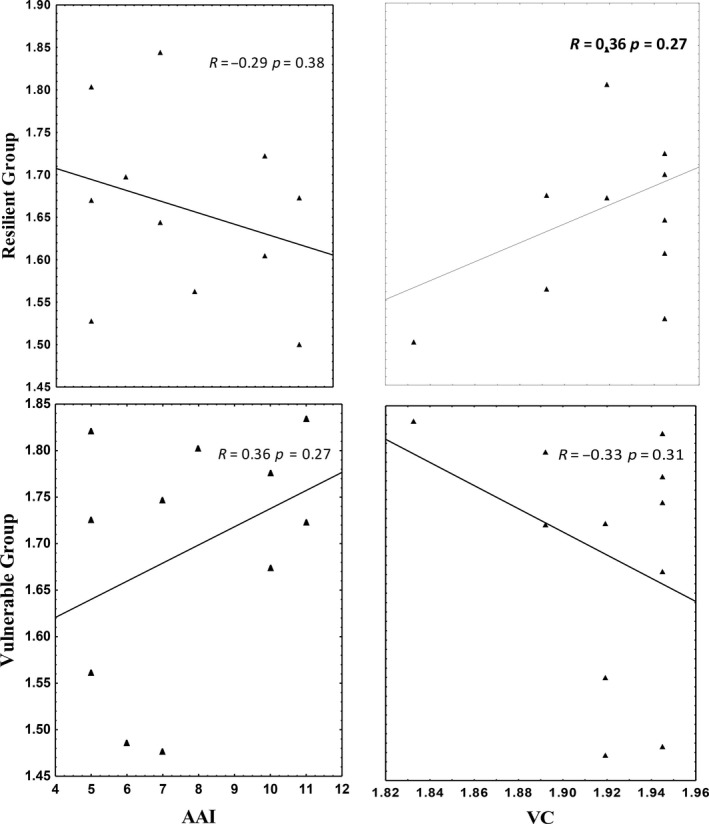
Linear regression between the relative abundance of the vulnerable group species, resilient group species and Anthropogenic Activity Index (AAI) and vegetation cover for the 11 surveyed hillslope seep wetland during summer

The relative abundance of species designated as resilient (RG) increased with increasing AAI, and the relationship was not statistically significant (Figure 5). During summer, the results indicated that the relative abundance of species designated as vulnerable increased with increasing AAI, while vulnerable species decreased with increasing VC, but the relationship was not statistically significant (Figure 6). The relative abundance of species designated as resilient (RG) decreased with increasing AAI, while it increased with increasing VC and the relationship was not statistically significant for either AAI or VC (Figure 6).

### 5.4 Percent relative abundance of species belonging to vulnerability groups across the site groups per season and interactions

**Figure 7:**
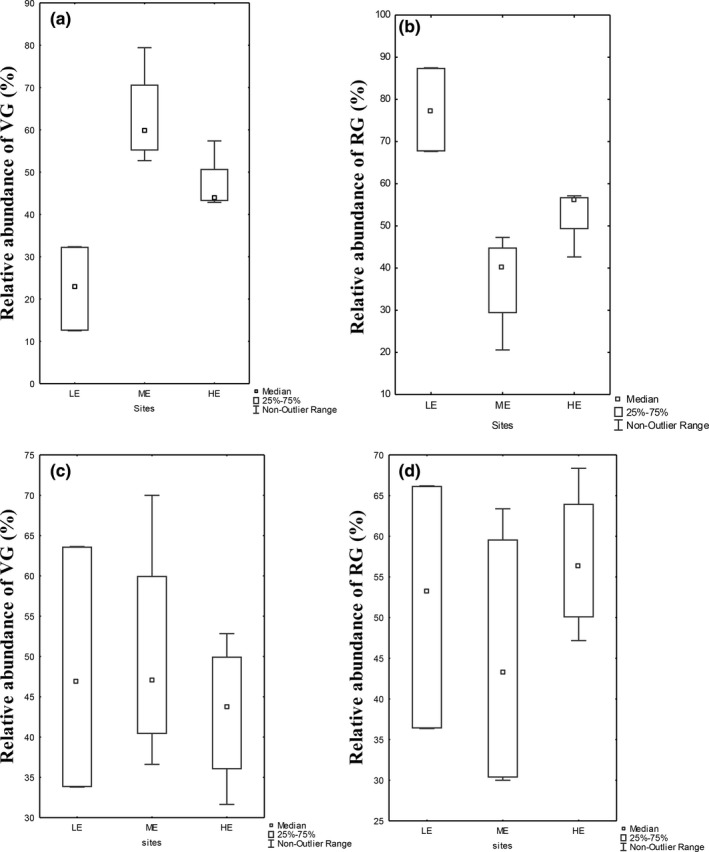
Relative abundance of the vulnerable group (VG) and resilient group (RG) across the site groups LE, ME, and HE for summer and winter. Sites: LE = less eroded, ME = moderately eroded, HE = highly eroded. Season: a and b = winter; c and d = summer. Abbreviation: groups: VG = vulnerable, RG = resilient

The box plot results showed that in summer, the relative abundance of species designated as vulnerable was most pronounced at the less eroded sites compared with highly eroded sites and there was a statistical significant difference between the sites in terms of the relative abundances (*p* < .05). The relative abundance of species designated as resilient was more evident at less and highly eroded sites with no statistical significant difference between sites (*p* > .05) (Figure 7). In winter, the results showed that the vulnerable species were most dominant in the moderately eroded sites compared with less and highly eroded sites, but no statistical significant difference between the sites was observed (*p* < .05). Looking at the seasonal pattern, the relative abundances of both vulnerable and resilient groups were higher in summer compared with winter.

### 5.5 Statistical associations between the individual plant species and the site groups

The Pearson's point‐biserial correlation analysis was run to assess the associations of individual plant species with the site groups (3LE, 4ME, and 4HE). Six distinct community types were observed (Table 6). The first community comprising six grass species was associated with the less eroded sites. The species in the community included *Panicum maximum*, *T. triandra*, *Alloteropsis semialata*, *Paspalum dilatatum*, *Wahlenbergia* sp., and *Commelina Africana*. Of these, only *T. triandra* showed a significant association with the less eroded sites (*p* < .05). Three of the associated species were considered to be vulnerable to disturbance, while the other three were considered to be resilient (Table 6). The second community consisted of two species, that is, *Ornithogalum* sp. and *Polygonum* sp. These species were associated with the moderately eroded sites. The association between *Ornithogalum* sp. and the moderately eroded sites was statistically significant (*p* < .05). Both species were classified as resilient (Table 6).

**Table 6:** Pearson point‐biserial correlation analysis of species association with site groups

Species	Site group 1	Site group 2	Site group 3	index	Coefficient	*p* value	VG
*Panicum maximum*	1	0	0	1	0.57	.2	V
*Themeda triandra*	1	0	0	1	0.91	**.031**	V
*Alloteropsis semialata*	1	0	0	1	0.73	.1	R
*Paspalum dilatatum*	1	0	0	1	0.91	.063	V
*Wahlenbergia* sp.	1	0	0	1	0.8	**.051**	R
*Commelina africana*	1	0	0	1	0.69	0.14	C
*Ornithogalum* sp.	0	1	0	2	0.83	**.04**	R
*Polygonum* sp.	0	1	0	2	0.7	.26	R
*Gerbera viridifolia*	0	0	1	3	0.7	.2	R
*Hemarthria altissima*	1	1	0	4	0.811	.87	V
*Hypoxis acuminata*	1	1	0	4	0.65	.4	V
*Taraxicum officinale*	1	1	0	4	0.59	.68	V
*Alepidea amatymbica*	1	1	0	4	0.78	.39	R
*Helichrysum aureonitens*	1	1	0	4	0.68	.64	R
*Knowltonia bracteata*	1	1	0	4	0.65	.41	V
*Eragrostis curvula*	1	1	0	4	0.86	.41	R
*Eragrostis plana*	1	1	0	4	0.93	.07	R
*Tristachya hispida*	1	0	1	5	0.53	.71	R
*Centella asiatica*	1	0	1	5	0.44	1	R
*Senecio speciosus*	1	0	1	5	0.63	.61	V
*Helichrysum nudifolium*	1	0	1	5	0.78	.27	R
*Conyza scabrida*	1	0	1	5	0.65	.4	V
*Digitaria erientha*	1	0	1	5	0.84	.09	V
*Miscanthus capensis*	0	1	1	6	0.66	.6	V
*Paspalum distichum*	0	1	1	6	0.8	.09	V
*Berkheya* sp.	0	1	1	6	0.61	.71	R
*Cymbopogon validus*	0	1	1	6	0.85	.24	R
*Mentha aquatica*	0	1	1	6	0.92	.074	V
*Senecio coronatus*	0	1	1	6	0.96	**.01**	V
*Richardia brasiliensis*	0	1	1	6	0.81	.54	R
*Eragrostis aspera*	0	1	1	6	0.61	.7	R
*Haplocarpha lyrata*	0	1	1	6	0.85	.24	R

Abbreviation: VG, species vulnerability group.

The numbers 1 and 0 under the respective site group columns indicate when a species is associated and not associated with the site group. Code: 1 (species associated with site group 1); 2 (species associated with site group 2); 3 (species associated with site group 3); 4 (species associated with site groups 1 and 2); 5 (species associated with site groups 1 and 3); 6 (species associated with site groups 2 and 3). Site group 1 (LE1, LE2, and LE3), site group 2 (ME1, ME2, ME3, and ME4), site group 3 (HE1, HE2, HE3, and HE4). Bold face indicates significant associations at *p* < .05.

The third community consisted of only one species, *Gerbera viridifolia*, which was associated with the highly eroded sites. The association was not significant. This species was classified as resilient.

The fourth community consisting of eight species was associated with the less and moderately eroded site groups. The species belonging to the community include *H. altissima*, *Hypoxis acuminata*, *Taraxicum officinale*, *Alepidea amatymbica*, *Helichrysum aureonitens*, *Knowltonia bracteata*, *Eragrostis curvula*, and *E. plana*. None of the species showed a significant association with the two sites combined. Four of these species were vulnerable, and four were resilient.

The fifth community comprising six species was concurrently associated with the less eroded and highly eroded site groups. The species in the community include *Tristachya hispida*, *C. asiatica*, *Senecio speciosus*, *Helichrysum nudifolium*, *Conyza scabrida*, and *D. erientha*. None of the associations between the species and the site groups was significant. Three of these species, *C. scabrida*, *S. speciosus*, and *D. erientha*, were considered vulnerable; three, *H. nudifolium*, *T. hispida*, and *C. asiatica*, resilient.

The sixth community comprising nine species was associated with site (less eroded and highly eroded groups). The species in the community include *Miscanthus capensis*, *Paspalum distichum*, *Berkheya* sp., *Cymbopogon validus*, *Mentha aquaticaa*, *Senecio coronatus*, *R. brasiliensis*, *Haplocarpha lyrata*, and *Eragrostis aspera*. Only *S. coronatus* showed a significant association (*p* < .05) with the site group. Five of these species were considered resilient, while four were vulnerable (Table 6).

Generally, the results showed that most species classified as vulnerable tend to be associated with the less disturbed sites, and some of those also appeared in moderately eroded sites, whereas the resilient species were most associated with moderately and highly eroded sites.

## 6 DISCUSSION

The TBA has been used widely to develop mechanistic models that predict the potential responses of biological assemblages to abiotic and biotic perturbations (Funk, Larson, Ames, & Butterfield, 2016). Some of these studies have been conducted on the impact of livestock grazing on vegetation patterns, based on the assumption that plant traits are useful in predicting species’ responses to grazing (Díaz et al., 2007). Traits have also been found useful for conservation studies and for identifying species vulnerability to land‐use changes (de Bello, Lepš, & Sebastia, 2005; Cingolani et al., 2005; Pillar, Duarte, Sosinski, & Fernando, 2009; Zheng, Li, Lan, Ren, & Wang, 2015). However, in South Africa, not much has been done in using traits as a mechanistic basis for understanding species–environment interactions. This is particularly true for grazing pressure in hillslope seep wetlands.

In the present study, plant species were grouped into vulnerable and resilient groups in relation to grazing pressure. It was then predicted that species belonging to the vulnerable group would be less dominant at the highly disturbed sites, compared with least disturbed sites. The results in summer corresponded with the prediction as vulnerable group species were more abundant in the less eroded sites compared with the highly eroded sites. The results also showed that the relative abundance of vulnerable species was higher in summer compared with the winter season. However, looking at the sites within winter, the results clearly indicated that most vulnerable species were more abundant at the moderately eroded sites compared with the less eroded sites, which may be attributed to the fact that some of the vulnerable species possessed mechanisms to resist disturbances. It could be that such mechanisms were not taken into account as part of the trait‐based methodology developed in the present study. And this clearly runs contrary to what was predicted for winter. Another factor could be that during winter, it is dry and species are grazed too short to be identifiable. The abundance of vulnerable species in moderately eroded sites might also be attributed to the fact that these sites are moderately impacted meaning that there is still a chance of vulnerable species to take place as the disturbance was not as much as highly eroded.

In terms of the association between individual species and the site groups, the Pearson point‐biserial correlation results indicated relative success for the approach developed, as species designated vulnerable were less associated with the highly disturbed sites. These species include *Panicum maximum*, *T. triandra*, *H. altissima*, and *P. dilatatum*. These species are generally highly palatable, tall with large leaves, thus exhibiting traits that reflect vulnerability (Dubey et al., 2011). Because the highly eroded sites in the study area are open access for livestock grazing, the lower association of these species with highly disturbed sites is attributed to high grazing pressure. Several studies have reported similar findings. For example, Díaz et al. (2007) demonstrated that grazing favoured species with resilient traits such as annual over perennial species, short plants over tall ones, prostrate over erect plants, stoloniferous and rosette architecture over tussock architecture. Cingolani et al. (2005) reported that short species with high SLA were abundant in most intensively grazed areas. Jones et al. (2010) found that tall and medium‐height species decreased with grazing intensity. The results indicate that resilient species show no pattern across sites, because they are able to survive in all wetlands, since they have traits that recover quickly after a grazing disturbance. Nevertheless, the resilient and vulnerable species largely coexisted in the less eroded sites, which may be due to niche partitioning and efficient utilization of resources by both groups of species, and thus the higher species diversity recorded in the less eroded sites. The results of this study suggest that when species possess traits that are resilient, they tolerate grazing pressure better than species that possess traits that are vulnerable. Therefore, management and sustainable conservation of hillslope seep wetlands may benefit from a TBA. Further, the predicted responses of vulnerable species group to disturbances showed that vulnerable species decreased with increasing AAI, implying that disturbance had an impact on the community pattern of the vulnerable species.

Regarding the degree of disturbance and grazing pressure in the studied hillslope seep wetlands, AAI results indicated significant differences between the three site groups and between the two seasons. AAI results showed that the less eroded sites were least disturbed compared with the highly eroded sites. The less eroded sites were on privately owned lands, which may have contributed to reduced grazing pressure as these sites were relatively well managed. By contrast, the moderately and highly eroded sites were in communal areas. A study conducted by Bella, Collins, and Jordaan (2018) indicates that communal wetlands were in poor ecological status while wetlands in privately owned lands were in excellent or good ecological status. Bella et al. (2018) indicated that overgrazing was a contributing factor to the poor ecological condition of wetlands in communal areas. The results of the present study are thus in agreement with those of Bella et al. (2018).

The VC indicated no statistically significant differences between the sites during the winter season. The observed result may be attributed to method used in assessing VC in this study. The method involves subjectively categorizing cover using scores that have been established. Similar observations that criticize the visual assessment of cover for being too subjective, since it is strongly dependent on the person who makes the observation, and can be quite variable have been also mentioned by (Damgaard, 2014). Point‐intercept method is regarded as an alternative more objective method for VC; however, this approach is not relevant for measuring the abundance of rare species and has been shown to underestimate species richness (Bråkenhielm & Qinghong, 1995).

The grazing pressure on the hillslope seep wetlands seemed to be highest during the winter season as indicated by the AAI and VC. A possible explanation is that in winter, hillslope seep wetlands offer about the only available green vegetation for grazing as the surrounding grasslands become less attractive for grazing due to dryness. Cattle may therefore be enticed to enter the hillslopes to obtain good quality grass for grazing (Hughes, McKergow, Tanner, & Sukias, 2013). These results agree with those of Wondie (2018) who reported wetlands that were highly degraded by overgrazing, particularly during the dry season.

## 7 CONCLUSION

A TBA was developed using a combination of multiple traits. Plant species were then classified into vulnerable and resilient groups in relation to grazing pressure. Based on the results from the study, two concluding remarks can be drawn. First, the approach developed enabled accurate predictions of the responses of hillslope plant species to grazing pressure seasonally, but spatially, only for the summer season. Second, the predicted responses during the winter season across sites did not match the observed results, which could be attributed to the difficulty in accurate estimation of VC during winter. Overall, the approach developed here provides a general framework for applying the TBA and can thus be tested and applied elsewhere.

## CONFLICT OF INTEREST

None declared.

## AUTHORS CONTRIBUTION

Notiswa Libala involved in original idea, served, as the project's principal investigator, designed the research project, collected the data, edited the manuscript, analyzed the research data, and written the first draft of the paper. Carolyn G. Palmer financially supported, designed the research project, edited the manuscript, and involved in supervising. Oghenekaro Nelson Odume involved in project idea, designed the research project, collected the data, edited the manuscript, and involved in supervising.

## Data Availability

The data used for this manuscript are archived in Dryad database https://doi.org/10.5061/dryad.jm63xsj69.
